# Mapping single molecule sequencing reads using basic local alignment with successive refinement (BLASR): application and theory

**DOI:** 10.1186/1471-2105-13-238

**Published:** 2012-09-19

**Authors:** Mark J Chaisson, Glenn Tesler

**Affiliations:** 1 Department Secondary Analysis, Pacific Biosciences, 1005 Hamilton Rd, CA, Menlo Park, USA; 2 Department of Mathematics, University of California, San Diego, 9500 Gilman Dr, CA, La Jolla, USA

## Abstract

**Background:**

Recent methods have been developed to perform high-throughput sequencing of DNA by Single Molecule Sequencing (SMS). While Next-Generation sequencing methods may produce reads up to several hundred bases long, SMS sequencing produces reads up to tens of kilobases long. Existing alignment methods are either too inefficient for high-throughput datasets, or not sensitive enough to align SMS reads, which have a higher error rate than Next-Generation sequencing.

**Results:**

We describe the method BLASR (Basic Local Alignment with Successive Refinement) for mapping Single Molecule Sequencing (SMS) reads that are thousands of bases long, with divergence between the read and genome dominated by insertion and deletion error. The method is benchmarked using both simulated reads and reads from a bacterial sequencing project. We also present a combinatorial model of sequencing error that motivates why our approach is effective.

**Conclusions:**

The results indicate that it is possible to map SMS reads with high accuracy and speed. Furthermore, the inferences made on the mapability of SMS reads using our combinatorial model of sequencing error are in agreement with the mapping accuracy demonstrated on simulated reads.

## Background

The first step in a resequencing study is to map reads from a sample genome onto a reference, accounting for sample variance and sequencing error. An accurate and sensitive approach is to use Smith-Waterman [1] alignment; however, this is computationally infeasible for mapping to nearly any genome. Instead, methods have been created using heuristics and data structures that are appropriate for rapid mapping of the type of read considered. For example, reads produced by Sanger sequencing that are highly accurate and nearly 1000 bases long are successfully mapped using hash-based methods such as MEGABLAST [2], cross_match (Green P., http://www.phrap.org, *unpublished*), and BLAT [3]. These methods are too inefficient to map read sets from next generation sequencing (NGS) instruments by Illumina (San Diego, CA, USA) and Life Technologies (Carlsbad, CA, USA), since they contain hundreds of millions of short reads. Instead, methods such as Bowtie, Bwa, and Soap2 are used [4-6]. These are based on querying the Burrows-Wheeler Transform Full-text Minute-space index (BWT-FM) [7] of a genome. They are able to rapidly align reads when there is little variation between the read and the genome.

Sequencing methods based on single molecule sequencing (SMS) also produce large datasets that have high computational demands for mapping. SMS datasets do not have the length limitations of NGS or Sanger sequencing, but have a higher number of errors, and the errors are primarily insertions and deletions rather than substitutions. Thus, mapping methods created for NGS sequencing do not extend well to SMS reads. A recent study using the PacBio*RS* platform [8] included a large number of reads over 10 kilobases long. As reads become longer, the computational problem begins to resemble the whole genome alignment (WGA) problems that were examined when multiple mammalian genomes were sequenced [9-11]. The problem arises of how to align long (many kilobase) reads with moderate divergence from the genome (up to 20% divergence, concentrated in insertions and deletions) at the speed and sensitivity that NGS alignment methods operate.

**Figure 1:**
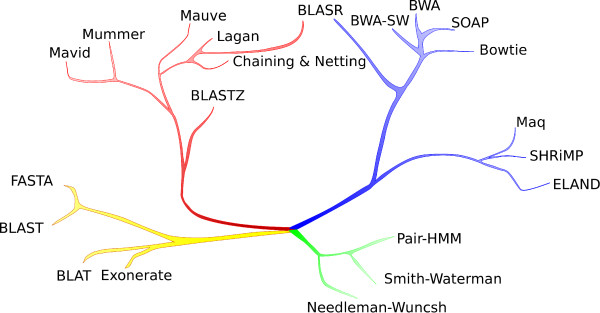
**An illustration of relationships between alignment methods.** The applications / corresponding computational restrictions shown are (green) short pairwise alignment / detailed edit model; (yellow) database search / divergent homology detection; (red) whole genome alignment / alignment of long sequences with structural rearrangements; and (blue) short read mapping / rapid alignment of massive numbers of short sequences. Although solely illustrative, methods with more similar data structures or algorithmic approaches are on closer branches. The BLASR method combines data structures from short read alignment with optimization methods from whole genome alignment.

Many alignment methods in similar application areas share related algorithmic approaches or data structures that are tailored to optimize the particular targeted application. The relationship between many existing alignment methods [1,3-5,10-23] is qualitatively illustrated in Figure 1. We present an approach, Basic Local Alignment via Successive Refinement (BLASR), which maps reads using coarse alignment methods developed during WGA studies, while speeding up these methods by using the advanced data structures employed in many NGS mapping studies.

Advances in isolation and detection of single molecules and reactions have enabled SMS methods [24-26]. These SMS methods monitor processes in real time. The PacBio*RS* instrument produces reads by detecting which fluorescently labeled nucleotides are incorporated into a DNA chain as a template sequence is replicated by DNA polymerase. Other SMS methods have been proposed using detection of cleaved bases that pass through a protein nanopore [25], and identifying bases that have translocated through a nanopore fabricated in a graphene membrane [27]. In the case of the PacBio*RS* sequencing, a missing or weak signal of nucleotide incorporation results in a deleted base, and nucleotides that give fluorescence signal without being incorporated lead to insertions.

We propose aligning SMS reads with high indel rates to genomes as follows. First, find clusters of short exact matches between the read and the genome using either a suffix array or BWT-FM index [7]. Then, perform a more detailed alignment of the regions where reads are matched to assign the alignment. To investigate the feasibility of doing this in the human genome, we need to determine two metrics: (1) the number of matches of minimal length expected to exist between a read and the genome at a given sequencing accuracy and read length, and (2) the number of false positive clusters the read is expected to have elsewhere in the genome. If the chances of finding a match between the read and the genome are low, or if there are many regions a read may map to incorrectly with high identity, our proposed approach would not be feasible. For a particular read length and accuracy, we present a method to determine the probability that the read contains a sufficient number of anchors to map; this method is based on counting integer compositions. We next examine the repeat structure of the human genome to determine how difficult it is to map to due to the repetitive nature of the genome. Rather than defining repeat content as the amount of sequence sharing high percent identity, we measure a different similarity metric on the human genome, the *anchor similarity*, where sequence similarity is measured as the number of shared anchors between the two sequences from the genome. We find that there are both a high number of expected matches between the read and the genome, and few false positive clusters of matches of the same size elsewhere in the genome, indicating that the proposed approach is feasible for mapping reads to the human genome.

We implemented our method in a program called BLASR (Basic Local Alignment with Successive Refinement), which combines the data structures used in short read mapping with alignment methods used in whole genome alignment. A BWT-FM index or suffix array of a genome is queried to generate short exact matches that are clustered and give approximate coordinates in the genome for where a read should align. A rough alignment is generated using sparse dynamic programming on a set of short exact matches in the read to the region it maps to, and a final detailed alignment is generated using dynamic programming within an area guided by the sparse dynamic programming alignment.

## Results and discussion

Our results are broken down into two sections; in the first, we examine characteristics of PacBio*RS* reads, and present theory on how these sequences contain matches that may be used to anchor alignments to the genome. In the next, we present a practical comparison of alignment methods on PacBio*RS* sequences.

### Mapping feasibility

Our strategy to map SMS reads is to locate a relatively small number of candidate intervals where the read may map and then use detailed pairwise alignments to determine the best candidate. The candidate intervals may be found by locating all exact matches between the read and the genome, and then finding dense clusters of exact matches (anchors) in spans of similar length and the same (or reverse complement) order and orientation in both the genome and read, as described in detail in Methods. The feasibility of the method depends on the balance of having enough anchors to detect the correct interval to align a read to, vs. having so many anchors that clustering takes a prohibitive amount of time.

**Figure 2:**
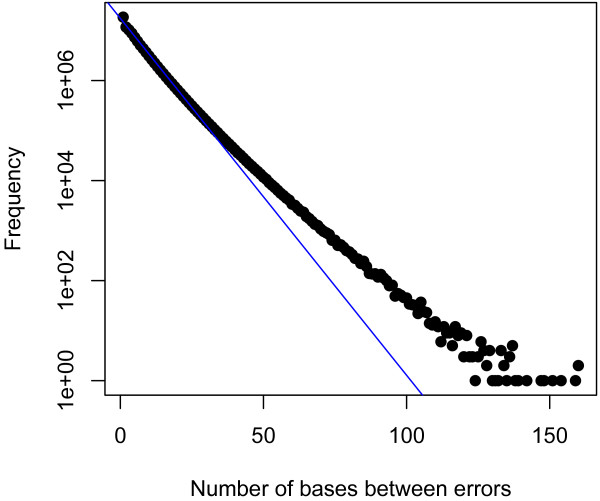
**The distribution of lengths of error-free segments of reads.** The line fitted to the points weighted by frequency has slope −0.071, corresponding to a geometric distribution with parameter 0.848, in close agreement with the 84.5% accuracy of the dataset used. Over 95% of segments are of length 20 less.

One approach to limiting the number of anchors is to limit to a set of anchors of low multiplicity in the genome; this is commonly done by using longer anchors. When the sequencing error rate is *ρ*per position, without positional bias, the average length of an exact match is $\frac{1}{\rho }-1$ bases. For *ρ* = 0*.*15, the average length is $\frac{1}{\rho }-1\approx 5.67$. Every word of length 5 occurs on average over 3 million times in the human genome, far too many times to be suitable as an anchor for rapid alignment. Fortunately, the condition that a sequencing error occurs precisely every ⌊1/*ρ*⌋ bases is worst-case, and is exceedingly rare: for a sequence of length *L* with roughly *E* = *ρL* sequencing errors, this only happens with probability on the order of 1/LE. Rather than focusing on the average case, it is more informative to consider the distribution of runs of error-free sequences; for a uniform distribution of errors across a read, this is a geometric distribution. To look at the empirical distribution of error-free sequences, a sample of reads from *Escherichia coli* sequenced by a PacBio*RS* was aligned back to the reference. The resulting distribution of spans of error-free sequences is shown in Figure 2, and closely follows the geometric distribution for over 95% of the data.

We may model SMS sequencing as a process that generates a series of error-free words with a geometric length distribution, each separated by a single erroneous base. With this model, it is possible to determine how many words must be sequenced until there is a high probability that a word of length *K* or greater (suitable for use in anchoring an alignment) has been sequenced. Denoting the length of a word as *W *, Pr{*W* = *K*} = (1 − *ρ*)^*K*^*ρ*, and Pr{*W* ≥ *K*} = (1−*ρ*)^*K*^, where *K*≥0. In order to have a probability of 1 − *ε*that a word of length *K* or greater is sequenced without error, *t* words must be sequenced, where $t=\frac{\log(\varepsilon )}{\log(1-{\left(1-\rho \right)}^{K})}$. The **waiting length** is the corresponding number of bases for *t* words, each followed by one incorrect base. The waiting length is

$$\begin{array}{ccc} t\left(1\;+\;\overset{K-1}{\underset{i=1}{\sum }}i\text{Pr}\left(W\;=\;i|W\;<\;K\right)\right)\; & \;= & t\left(1+\overset{K-1}{\underset{i=1}{\sum }}i\frac{{\left(1-\rho \right)}^{i}\rho }{\left(1-{\left(1-\rho \right)}^{K}\right)}\right)\\ & \;= & t\left(\frac{1}{\rho }-\frac{K{\left(1-\rho \right)}^{K}}{1-{\left(1-\rho \right)}^{K}}\right)\;.\; \end{array}$$

**Figure 3:**
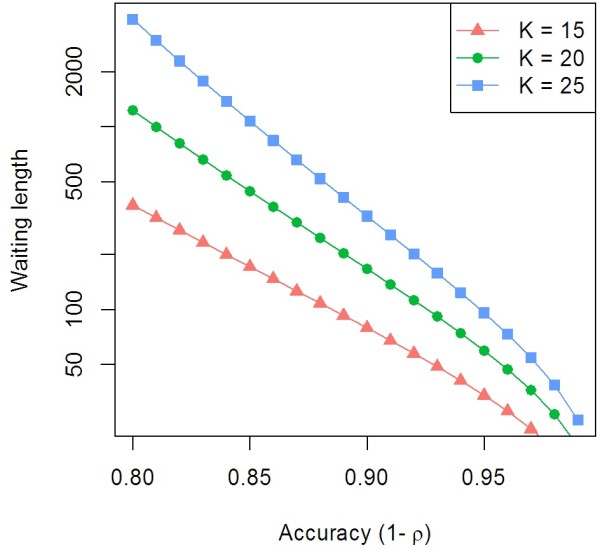
**Waiting length to sequence a word of length*≥k*at*ε = 0.05*.** The waiting lengths to sequence a word of length ≥ k at ε = 0.05 at varrying accuracy. This gives an estimate of the number of bases required to sequence before having an error free stretch that may serve as an alignment anchor.

The waiting lengths for words of size 15, 20, and 25 are shown for *ε* = 0*.*05 and varying *ρ*in Figure 3. We refer to error-free sequences of length *K* or greater as **anchors**.

Other alignment methods such as Gapped BLAST [28] and BLAT [3] have shown that it is useful to initiate alignments at pairs of anchors. The waiting lengths may be used to compute the length of read required to be certain of having at least N anchors. Instead of using waiting lengths, it is possible to directly compute the probability of sequencing a certain number of anchors when the error rate is known. We do this with a model that approximates all errors as point mutations on a scan across a template. Given a fixed template length L, a minimal anchor length K, a number of errors M, and a number of anchors N, define **NumConfigurations*(M,N,K,L)*** as the number ways to distribute the positions of M errors when reading from the template such that there are at least N maximal substrings of length ≥ K not interrupted by error. In Appendix 1, we compute this using generating functions, allowing us to apply the result across the read lengths and error profiles found in SMS sequencing. Weese et al. [29] considered a similar problem for short reads and low error rates, and set bounds for filtering alignment hits in a q-gram based mapping method by using a dynamic programming approach.

**Figure 4:**
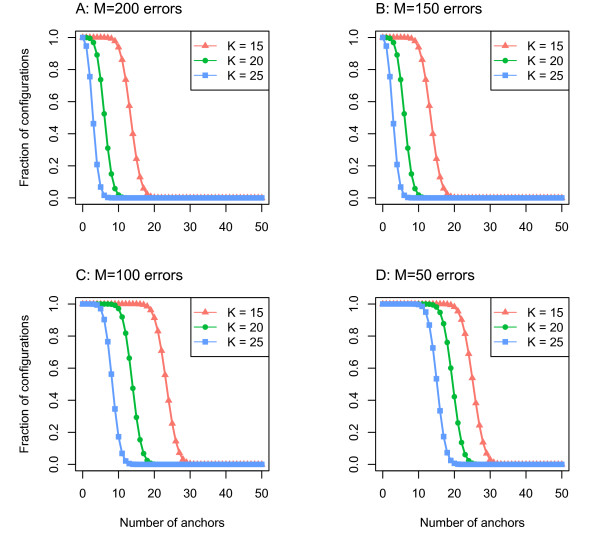
**Values for**NumConfigurations(M,N,K,L)/LM**for parameters similar to SMS sequencing.** The fraction of configurations allowing at least N anchors of length 15, 20, and 25 for N between 0 and 50 are shown for a 1000 base read when placing (**A**) 200, (**B**) 150, (**C**) 100, and (**D**) 50 errors.

Assuming all permutations of errors are equally likely, NumConfigurations(M,N,K,L)/LM gives the probability of sequencing at least N anchors. We computed this probability for the parameters L = 1000, and K = 15, 20, and 25, to study the number of anchors to use for mapping. The results are shown in Figure 4 for M = 200, 150, 100, and 50, corresponding to read accuracies of 80%, 85%, 90%, and 95%. At an accuracy of 85%, nearly all configurations have at least 10 anchors of length at least 15. This indicates that with minimum anchor size K = 15, one would would expect to find at least 10 anchors at the correctly mapped interval in the genome.

When a read is sampled from a repeat in the genome, there are likely to be many dense clusters of anchors mapping the read across the genome. Assuming the repeat is divergent, it is necessary to perform a detailed alignment (Smith-Waterman) to all intervals containing dense clusters of anchors in order to distinguish the correct mapping location from other repeats. For copies of a repeat such as Alu or LINE in the human genome, the computational demands are too prohibitive to align the read against all instances of the repeat. On the other hand, if only a limited number of mapped locations are aligned in detail, the chance of finding the correct location is small. The similarity of repeats in a genome is typically defined by percent identity from a pairwise alignment of the two sequences [30]. However, sequences that have a high percent similarity may not share many long stretches of exact matches, which is how they are compared when using anchor-based mapping. To characterize repeats with respect to anchor-based mapping, we introduce an alternative metric: the **anchor similarity** of two sequences is the maximum number of fixed-length, non-overlapping, ordered anchors, shared between two sequences, with certain constraints on anchor spacing. If the anchor similarity is S, we also say the two sequences are ***S*-similar**, and ***≥S*-similar** when two sequences have anchor similarity that is at least S. Using fixed-length anchors simplifies the presentation, although the BLASR method uses variable length anchors. Anchor similarity requires two parameters: K, the minimum anchor size; and δ, the indel rate, which may change the spacing between anchors. The constraints reflect the spacing one would expect between anchors of a read with indel errors and a genome. For example, consider a sequence that contains anchors at coordinates a and b, matching anchors at coordinates a^′^ and b^′^in another sequence. If the ratio of the gaps between anchors is bounded by $1-\delta \leq \frac{b-a}{b^{\prime }-a^{\prime }}\leq 1+\delta$ (consistent with the indel rate), then a and b may be included in the count for the anchor similarity of the two sequences. Further details on computing anchor similarity are given in the Additional file 1: Text S1, Section 1.1.

**Figure 5:**
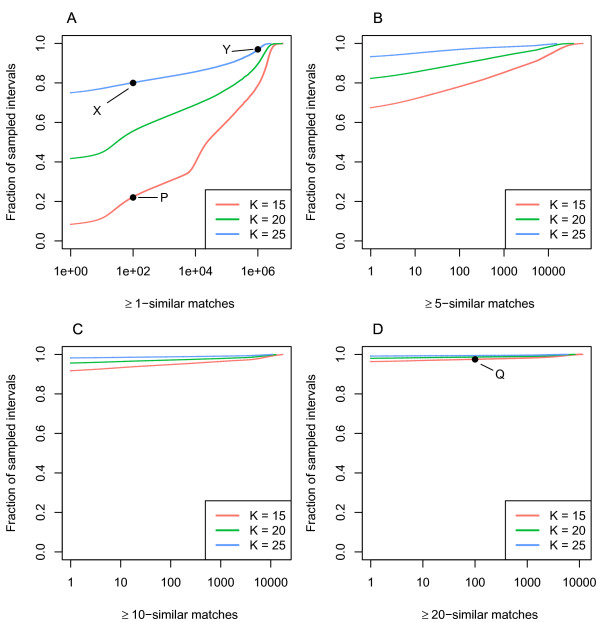
***≥S*-similar sequences measured in the human genome.** 1 million query intervals, each 1000 bases long, were randomly sampled from the genome. Each query interval was searched against the human genome to determine the number of non-overlapping 1000 base intervals in the genome that are ≥*S*-similar to the query. The cumulative distribution for the number of target intervals that are (**A**) ≥1-similar, (**B**) ≥5-similar, (**C**) ≥10-similar, and (**D**) ≥20-similar to these 1 million query intervals, is shown. Each panel uses minimum anchor lengths *k* = 15, 20, and 25 and indel rate *δ* = 0*.*15. From this, one may interpret the number of intervals that must be searched when mapping a read using anchors. For example, when mapping with a minimum of a single 25 base match, 80% of the queries match to 100 other intervals in the genome with at least one one 25 base match (point X). On the other extreme, the top 3% of queries map to over 1 million other with at least one matchpoint Y), due to the high repeat content of the genome. This indicates that 80% of sequences may be correctly mapped to the human genome using a single 25 base match by only searching 100 100 candidates, however for full sensitivity many more candidates must be searched. Points P and Q show a contrast of the fraction of intervals that have 100 or fewer matches in the genome when matching using 1 or more anchors versus 20 or more anchors, for an anchor length of 15. Only 20% of the samples are limited to 100 or fewer additional matching intervals with at least 1 anchor (point P), and 97.5% of the samples have 100 or fewer matches when requiring at least 20 anchors in a match (point Q).

To characterize the repetitiveness by anchor similarity of sequences in the human genome, we took a sample of 1 million random intervals of length L=1 kb in the genome, and computed anchor similarity of each interval with all other intervals up to length (1 + δ)L = 1150 (assuming an indel rate δ = 0.15) in the rest of genome. We used anchors of lengths 15, 20, and 25. For each interval and anchor length, a histogram is generated for the number of times ≥S-similar intervals are found in the genome. A hypothetical sample sequence with K = 15 may have 50 thousand ≥1-similar intervals in the genome; one thousand ≥2-similar intervals; one hundred ≥3-similar sequences; ten ≥4-similar sequences; and one ≥5-similar sequence. This results in one million histograms (for each anchor length). To summarize these, we examined the cumulative distribution of values of all histograms for ≥1, ≥5, ≥10, and ≥20-similar sequences, as shown in Figure 5.

We compared the distribution of values of anchor similarity from the human genome with values of NumConfigurations(M,N,K,L)/LM to see how the mapability of sequences compares to the expected distributions of matching anchors. Reads from intervals of a genome that have low anchor-similarity to the rest of the genome are likely to have few spurious matching clusters and are thus likely to be uniquely mapped. Conversely, a read sampled from an interval that has high anchor-similarity with many other intervals likely has many clusters of matches to the genome. Figure 5 shows an estimate of the number of intervals that must be searched when using anchor-based seeding to gain a certain degree of sensitivity of finding the true match. For example, when requiring only one or more matches of length 15 to find an interval, 22% of the sequences have up to 100 matching intervals in the genome (Figure 5A, point P). If instead 20 or more matches were required in order to find an interval, 97% of the regions of the regions sampled have up to 100 matching intervals in the genome (Figure 5D, point Q). The combination of the values of NumConfigurations(M,N,K,L)/LM and intuition for the feasibility of mapping sequences at various error rates in the human genome. From Figure 4, for reads sequenced at 85% accuracy, it is very likely there are least 8 anchors of there are least 8 anchors of length 20 or greater in any read. The green points in C show the number of matching intervals when using a similar set of parameters: at least 10 anchors of length 20. Importantly, 95% of the samples match uniquely in the genome.

**Figure 6:**
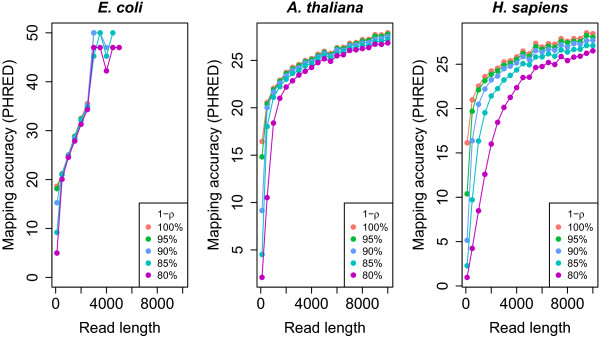
**The mapability of simulated sequences from the*E. coli*,*A. thaliana*, and human genomes.** Mapping accuracy is shown on a Phred scale ( $-10\log\frac{\text{missing}+\text{mismapped}}{\text{total}}$) for all three plots. Reads were simulated with base accuracies 1−*ρ* = 80*%*, 85%, … , 100%. In the fraction *ρ*of positions that are erroneous, we simulated 10% substitutions, 62% insertions, and 28% deletions. Missing values have no mismapped reads.

To gauge the mapability of sequences to various genomes, we simulated reads from *Escherichia coli*, *Arabidopsis thaliana*, and human, for read lengths that vary from 100 to 10000 bases, and error rates from 20% down to 0%. We mapped them back to their reference genomes with BLASR (see The results are shown in Figure 6. We note for mapping to the human genome, while it is difficult to have precise predictions on the mapability of sequences, the results are in agreement with the inferences drawn from the distributions of number of anchors and anchor-similarity measures. For example, 95% of 1000-base reads from the human genome simulated with a 15% error rate map to the correct location in the genome.

As shown in Figure 4B, a read with a 15% error rate has a 97% chance of having 10 anchors of length 15 or more. The anchor similarity corresponding to these reads uses parameters *δ* = 0*.*15,*L* = 1000, and *k* = 15, and is shown by the red curve in Figure 5A. Over 90% of the sampled intervals only have one location with at least 10 anchors of length 15, indicating they map uniquely under this repeat under this repeat metric. The other two genomes, *E. coli*, and *A. thaliana*, are shown for

### Mapping benchmarks

We generated three datasets for evaluating mapping speed and accuracy of different aligners on SMS reads (see Table 1). For all *E. coli* datasets, reads were aligned to the genome of an isolate of the O104:H4 strain (doi: 10.5524/100001). The source reads are available at http://bix.ucsd.edu/projects/blasr. Performance was measured additionally with both BLAT and the BWA-SW aligners [18]. BWA-SW was the first mapping method written that used both the BWT-FM index used in short read mapping and methods that allow mapping long reads with indel error. This method is very compact (under 5 GB of memory for human genome alignments), and very sensitive to mapping reads with indel error, as compared to other existing methods. Other methods that were tested either did not run or produced insufficient results. This may be expected, as these methods are highly optimized for other types of data that is either short read or whole genome sequences. Of the programs that did not run, Soap2 and Lagan crashed, while Bowtie did not accept the read input due to read length, and the mapping sensitivity was low on reads truncated to the maximum allowed length. The Mosaik (Strömberg M., http://bioinformatics.bc.edu/marthlab/Mosaik, *unpublished*), Mummer, and RazerS methods did execute, however the first two could only align to one chromosome of the human genome at a time due to space limitations, and were orders of magnitude slower than either BLASR or BWA-SW while finding very few hits. Finally, the RazerS method was only tested on *E. coli* reads, and found few hits across all tested parameters. Because of the low mapping sensitivity, these methods were excluded from benchmarking results. The BLAT method is included as a reference for comparison to methods optimized for mapping Sanger sequences, though it is slower and less sensitive than both BLASR and BWA-SW.

**Table 1:** Datasets used in benchmarking

**Dataset**	**Description**
*E. coli*-PacBio*RS*	*E. coli* O104:H4 sequenced at 48× coverage by the
	Pacific Biosciences-*RS* sequencer.
*E. coli*-simulated	50× coverage of reads simulated from *E. coli* O104:H4.
*H. sapiens*	100 MB of reads simulated from the human genome.

**Figure 7:**
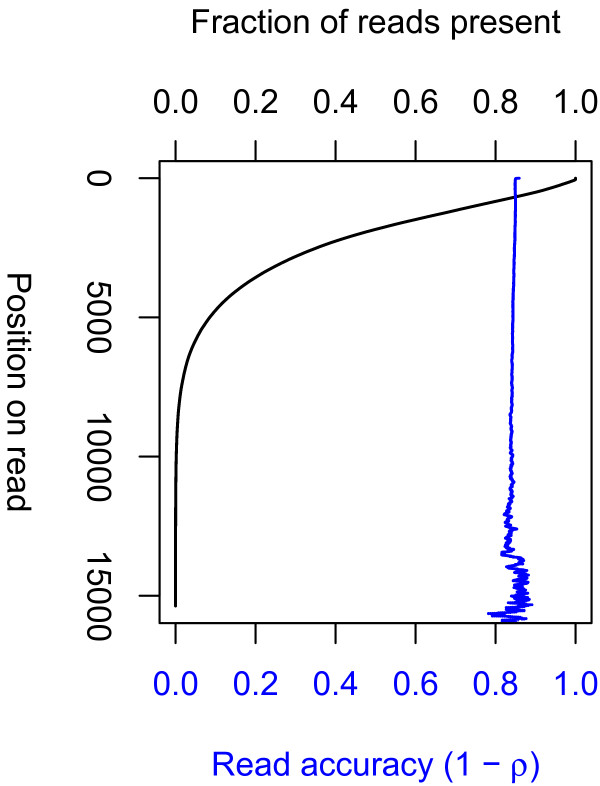
**Statistics of reads from*E. coli*O104:H4 produced by the PacBio*RS*sequencing platform.** (*Black*) The fraction of reads with length at least *x*. This is roughly the survival curve of an exponential distribution. (*Blue*) The fraction of reads (of length at least *x*) that are correct at position *x*. Accuracy is nearly position independent, so the blue curve is roughly the constant 1−*ρ*, where *ρ*is the error rate per position.

The *E. coli*-PacBio*RS* dataset contains 123,246 reads comprising 261.7 M bases after filtering, with lengths and error rate shown in Figure 7 (Short Read Archive accession numbers SRR305922, SRR305923, SRR305924, and SRR305925). The reads contain 10.7% insertion, 4.3% deletion, and 0.9% substitution error, though the details are sensitive to alignment penalty summary of the mapping statistics from each of the three programs is shown in Table 2. All programs were executed on a single core of a 2.9 GHz Xeon processor. The parameters used for each program are given in Additional file 2: Table S1.

**Table 2:** **A comparison of the BLASR, BWA-SW, and BLAT methods on*E. coli*reads**

**Method**	**Number of aligned reads**	**Number of aligned bases**	**Run time**
BLASR-SA	94057	230.8 M	20m 54s
BLASR-BWT	94527	230.1 M	33m 57s
BWA-SW	97729	132.4 M	434m 5s
BLAT	99530	181.7 M	4724m 40s

Each method was used to align 48× coverage of reads from *E. coli* O104:H4. BLASR-SA uses a suffix array index of the genome, while BLASR-BWT uses a BWT-FM index of the genome.

To test the sensitivity and specificity of mapping, reads were simulated using an empirical model (described in Additional file 1: Text S1, Section 1.2) based on the measurement of error rates from reads aligned to *E. coli*. The results are shown in Table 3. The methods are largely in agreement on the reads that are correctly mapped, as well as in the number of bases from every read, and BLASR is marginally faster. The slight differences in mapping statistics between BLASR-SA and BLASR-BWT are due to implementation differences in the order anchors are generated: using a suffix array, sequences are searched left to right, but for a BWT-FM index, sequences are searched right to left. One difference between BLASR and BWA-SW is that BWA-SW often produces several short alignments of possibly overlapping substrings of a read rather than one contiguous alignment. We consider the number of bases mapped by BWA-SW as the sum of uniquely mapped bases from each read. Usually this does not affect mapping and consensus quality, but occasionally there are subsequences from reads that are incorrectly mapped while the rest of the read is mapped correctly.

**Table 3:** A comparison of the BLASR, and BWA-SW methods on simulated reads

**Method**	**Correctly mapped**		**Incorrectly mapped**		**Skipped**	**Runtime**	**Memory **	
	**reads**	**bases**	**reads**	**bases**	**reads**		**footprint**	
***E. coli***								
BLASR-SA	108789	266.5M	229	0.38M	3766	48m 18s	202 MB	
BLASR-BWT	108795	265.3M	259	0.45M	3604	59m 39s	46 MB	
BWA-SW	111192	261.9M	1835	0.91M	3005	223m 57s	190 MB	
***H. sapiens***								
BLASR-SA	41726	102.3M	1074	1.89M	413	92m 26s	14.7 GB	
BLASR-BWT	41582	101.7M	1159	1.75M	472	53m 26s	8.1 GB	
BWA-SW	40381	96.3M	292	1.16M	1554	105m 24s	4.2 GB	

Reads are simulated from *E. coli* and *H. sapiens* with length and accuracy parameters modeled from real reads from *E. coli*. Skipped reads are either marked as filtered in the SAM output, or missing from the output.

**Figure 8:**
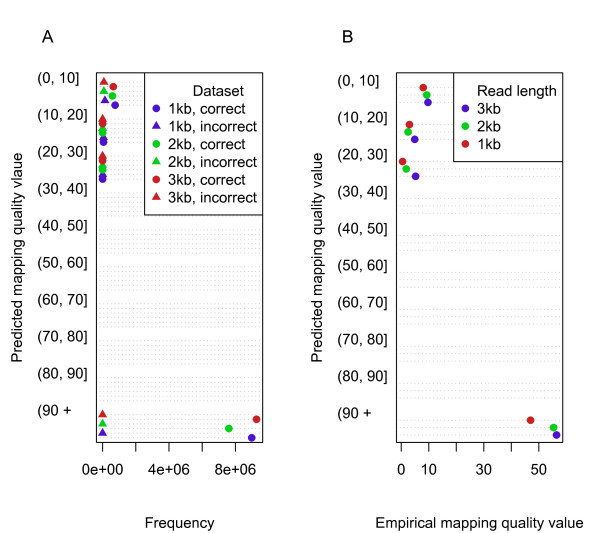
**Mapping quality values of reads simulated from the human genome.** (**A**) The frequency of quality values for alignments of 10^6^simulated 1000, 2000, and 3000 base sequences from the human genome. (**B**) The empirical mapping quality values of the alignments.

In addition to the information encoding the alignment, BLASR produces a mapping quality value for every alignment. This value represents the PHRED scale probability that the coordinates the read is aligned to in the genome are incorrect, similar to the mapping quality values produced by Maq [20]. To test mapping quality values, we created three datasets of 10M simulated reads sampled from the genome with fixed read lengths of one, two, and three kilobases each. Errors were added to the reads using the empirical read simulator (Additional file 1: Text S1, Section 1.2). For each mapped read, we classified it as correctly and incorrectly mapped, allowing a measurement of accuracy of mapping quality value. The frequency of computed mapping quality values are shown in Figure 8A. The mapping quality values are largely binary, owing to the fact most reads contain sequences that align uniquely to the genome. The empirical mapping quality values are shown in Figure 8B.

## Conclusion

Methods to produce reads through single molecule sequencing were mostly theoretical a decade ago and are now produced in high throughput on an industrial platform. The different characteristics of the sequences produced by SMS relative to Next Generation sequencing (sequences several orders of magnitude longer than previous technologies, at the expense of a higher error rate concentrated in insertions and deletions), require new computational techniques to be efficiently analyzed. Here, we addressed the problem of mapping SMS reads to a reference genome by first examining the feasibility of mapping SMS reads, and then by benchmarking our new alignment method on reads produced by the PacBio*RS* instrument. The source code is available under the BSD license at https://github.com/PacificBiosciences/blasr and is the default alignment method available to all running the PacBio*RS*.

There are many emerging problems for processing SMS sequences. As the length of the reads produced by SMS increases, the computational problem resembles whole genome alignment more than the read mapping problem. This increases the need to have methods that accurately detect structural rearrangements covered by single reads. Furthermore, with the inevitable exponential increase in sequencing throughput, the current methods will not be sufficient to align SMS reads without a large amount of time or computational resources, and further algorithmic improvements will be necessary. We did not address the issue of using multiple sequence alignment to produce a consensus sequence or variant calls. It has been shown that the additional information one may gain by observing the signal from single-molecule events in real time may indicate DNA modifications such as methylation [25,31]. Thus, methods that produce consensus calls from SMS sequences may reveal more information about the sample sequence if this extra information is used. We aim to address many of these problem in subsequent iterations of the BLASR method.

## Methods

**Figure 9:**
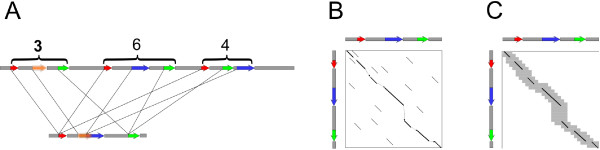
**Overview of the BLASR method.** (**A**) Candidate intervals are found by mapping short, exact matches as shown by colored arrows. Either a suffix array or BWT-FM index of the genome are used to find the exact matches. Intervals are defined over clusters of matches and are ranked; intervals with score 3, 6, and 4 are shown. (**B**) Matches scoring above a threshold are aligned using sparse dynamic programming on shorter exact matches. (**C**) Alignments that have a high-scoring sparse-dynamic programming score are realigned by dynamic programming over a subset of cells defined using the sparse dynamic programming alignment as a guide.

We use a *successive refinement* approach to map SMS reads. This approach operates in three phases: (1) detecting candidate intervals by clustering short, exact matches; (2) approximate alignment of reads to candidate intervals using sparse dynamic programming; and (3) detailed banded alignment using the sparse dynamic programming alignment as a guide, as shown in Figure 9. It is not until the third step that read base positions are assigned to reference positions.

### Detecting candidate intervals

The input to the BLASR method is a read *r* with nucleotides *r*_1_,…,*r*_*R*_; a genome *g* with nucleotides *g*_1_,…,*g*_*G*_; and a minimum match length, *K*. Other parameters that modify small details of mapping are introduced in their context later. We find all exact matches of substrings (of length at least *K*) from the read and the genome. An exact match of anchor *a* to the genome may be described by a triplet (Read(*a*), Genome(*a*), *l*(*a*)), where Read(*a*) is the start of the match in the read; Genome(*a*) is the start of the match in the genome; and *l*(*a*) is the length of the match. The set of all matches is A.

We use either a suffix array (SA) or BWT-FM index on the genome to query for exact matches, depending on time and space requirements. While some NGS alignment methods such as mrFAST and RazerS match using hash tables on fixed width words (*q*-grams) [29,32], the SA and BWT-FM index allow matching long exact matches if they exist, and also encode positions of shorter matches if a more sensitive search is required. The two data structures support the same queries: *c* = Count(*qt*), the number of times a query sequence *r* occurs exactly in a text *g*; and $P=\{p_{1},\ldots ,p_{c}\}=\text{Locate}(q,t)$, the starting positions of all instances of *r* in *g*. Without changing the computational complexity of these queries, they may be modified to answer equivalent queries for counts and locations of the longest common prefix (LCP) between a query and a genome. Let (*cl*) = COUNTLCP(*qt*) be the operation that finds the count *c* and length *l* of the LCP between *q* and *t*. We locate anchors by greedily finding matches slightly *shorter* than the LCP (specified by a parameter defaulting to 1 base shorter than the LCP) to increase sensitivity and avoid using an LCP that erroneously ends in a sequencing error. The minimum length anchor that is allowed is of length *K*, where *K* = 12 in most applications. To build A, we scan across all positions in a read *i* ∈ {1,…,*R* − *k*}; we compute (*cl*_*i*_) = COUNTLCP(*r*_*i*,…,*R*_*g*) and $P_{i}=\text{Locate}(r_{i,\ldots ,i+l_{i}-e},g)$; and then for all positions $p_{j}^{i}\in P^{i}$, we include in A a match *a* with Read(*a*)=*i*, $\left(\text{Genome}(a)=p_{j}^{i}\right)$, and *l*(*a*) = *l*_*i*_. We choose a parameter MaxCount, which specifies the maximum number of times we allow a match to appear to generate an anchor. We exclude positions mapped when $|P^{i}|>\text{MaxCount}$, or short matches when *l*_*i*_ < *k*.

Descriptions of the implementation and methods for the Count and Locate queries using suffix arrays are given in [33]. Similar descriptions for the BWT-FM index are in [7] and [4]. The CountLcp operation is about 1.5× faster using a suffix array than a BWT-FM index, in our tests searching the human genome and limiting the number of times an LCP occurs to 10,000; however, the space usage for the index on a human genome is 12.8 GB with a suffix array, vs. 4.8 GB in our implementation of a BWT-FM index. Our implementation of the Locate operation is faster for larger genomes using the BWT-FM index than the suffix array when using SIMD hardware optimization. Because either index is shared across many threads, the amortized space usage is modest for both data structures.

Once the set of anchors A is generated, we cluster anchors using global chaining [34]. To do so, we first sort A by position in the genome and then by position in the read. Next, clusters of anchors are found in intervals roughly the length of the read. For every anchor $a_{i}\in A$, a set $A_{i}$ is created with $A_{i}=\{a_{j}\in A:0\leq \;\text{Genome}(a_{j})+ł(a_{j})-\;\text{Genome}(a_{i})\leq R\}$. For every set $\left(A_{i}\right)$, we find a maximal subset (using global chaining) of anchors, $\left(C_{i}\subset A_{i}\right)$, that are not overlapping and are increasing in both Read(*a*) and Genome(*a*). For later use in evaluating the mapping quality value of a read, for each cluster, we record the sum of all ł(*a*) values for all anchors in $\left(C_{i}\right)$.

The clusters are assigned a frequency weighted score that is the sum $\left(\underset{a_{j}\in C_{i}}{\sum }\text{log}(1/\;\text{Freq}(a_{j}))\right)$, where Freq(*a*_*j*_) is the frequency of the sequence of *a*_*j*_ in the genome, and are ranked by this score. Only the top MAXCANDIDATES clusters are retained (typically 10). The original indexing of clusters by anchor position is replaced by indexing by rank of the frequency-weighted score. The subscript notation is dropped and rank of a cluster is indicated by the superscript. The remaining clusters are denoted $\left(C^{1},C^{2},\ldots ,C^{n}\right)$, where $\left(\text{rank}(C^{1})\leq \text{rank}(C^{2})\leq \ldots \leq \text{rank}(C^{n})\right)$, and *n* ≤ MAXCANDIDATES.

While limiting the number of clusters retained may miss alignments to repetitive regions, filtering clusters on this frequency-weighted score was shown to be highly discriminative in our tests.

### Refining alignments

Each cluster $\left(C^{i}\right)$ is used to define an interval to which the read is realigned and rescored using sparse dynamic programming (SDP) [35]. To help describe how the interval is defined, let *a*^FIRST^(*a*^LAST^) be the anchors with least (greatest) Genome(*a*) and Read(*a*) coordinates in $\left(C^{i}\right)$, ordered by position in genome and then by read. The anchors in $\left(C^{i}\right)$ frequently do not contain the first and last bases in the read, and the actual starting and ending positions of the read are unknown due to insertion and deletion error in the read. Considering *δ* to be the maximum insertion rate of the instrument, the starting position of the interval aligned from the genome is *s* = Genome(*a*^FIRST^) − (1 + *δ*)Read(*a*^FIRST^), and ending position *f* = Genome(*a*^LAST^) + (1 + *δ*)(*R*−(Read(*a*^LAST^) + *l*(*a*^LAST^))), of length $\left(l_{C}=f-s\right)$.

The read must be quickly aligned to a candidate interval, even if it is many tens of kilobases long. Similar to the method of anchoring the interval to the genome but on a smaller scale, a set of matches are found between the read and the candidate interval. The matches used in SDP are of a short fixed length, *K*^SDP^ (typically 8–11 bases). Let $\left(A^{\text{SDP}}\right)$ be the set of anchors of length *K*^SDP^ that are exact matches between the read and the genome interval *g*_*s*_,…,*g*_*f*_. Sparse dynamic programming finds the largest subset of anchors $\left(C^{\text{SDP}}\subseteq A^{\text{SDP}}\right)$ that are of increasing Read(*a*) and Genome(*a*) values.

The SDP alignment does not align all bases in a read, and so it is necessary to realign a final time using banded dynamic programming. For long reads with indels, the size of the band used to contain the entire alignment becomes prohibitively large. The set of anchors $\left(C^{\text{SDP}}\right)$ forms a guide for performing a banded dynamic programming alignment where the band follows the layout of the anchors in $\left(C^{\text{SDP}}\right)$, shown in Figure 9C. The subset of cells included from full $\left(R\times l_{C}\right)$ dynamic programming grid include a band of length *b*^SDP^centered about the diagonal where there are anchors, as well as a banded alignment of size *b*^drift^ between anchors where *b*^drift^ is the off-diagonal distance between adjacent anchors + *b*^SDP^.

In addition to the base sequences produced by the PacBio*RS*, each base in the read is also given three quality values (insertion, deletion, and substitution) and two alternative base calls (substituted base and deleted base). Let $\left(I\right)$, $\left(S\right)$, $\left(D\right)$ be the insertion, substitution, and deletion quality value arrays for a read, and $\left(Ŝ\right)$ and $\left(\hat{D}\right)$ be the deletion and substitution nucleotide arrays. We use these quality values to compute the score of each cell *s*_*i*,*j*_ in the dynamic programming matrix according to:

$$s_{i,j}\;=\;\min\;\left\{\;\;\begin{array}{c} s_{i-1,j-1}+\left\{\begin{array}{cc} 0 & \;\text{if}\;r_{i}\;=\;g_{j}\\ S_{i}\;\;\;\; & \;\text{if}\;r_{i}\;\neq \;g_{j},{Ŝ}_{i}\;=\;g_{j}\\ \text{MISMATCHPRIOR} & \;\text{otherwise} \end{array}\right)\\ s_{i-1,j}+I_{i}\;\;\;\;\;\;\;\;\;\;\;\;\;\;\;\;\;\;\\ s_{i,j-1}+\left\{\begin{array}{cc} D_{i}\;\;\; & \;\text{if}\;{\hat{D}}_{i}=g_{j-1}\\ \text{DELETIONPRIOR} & \;\text{otherwise.}\;\;\;\; \end{array}\right) \end{array}\right)$$

The MISMATCHPRIOR and DELETIONPRIOR are PHRED scaled penalties that reflect the global mismatch and deletion rates. In practice, MISMATCHPRIOR is 20 and DELETIONPRIOR is 15.

### Mapping quality values

Due to the repetitive nature of genomes, a read often maps with a high alignment score to many locations. It is informative to calculate the probability that the interval a read is mapped to by an alignment is the correct location in the genome. This probability may be interpreted as a *mapping quality* value $\left(Q\right)$ for an alignment, allowing downstream analysis such as variant calling to filter alignment by quality.

A Bayesian probability technique was presented in [20] to compute the mapping quality for short reads with base calling quality values. We present the formulation in [20] using the notation in this paper: we are given read *r* and a position *m* that it is mapped to in a sequence *g*. The *posterior mapping probability* that a read *r* is sampled from *m* is computed as

(1) $${\text{Pr}}_{s}(m|r,g)=\frac{\text{Pr}(r|g_{m,\ldots ,m+R-1})\text{Pr}(m)}{\sum_{i}\text{Pr}(r|g_{i,\ldots ,i+R-1})\text{Pr}(i)},\;$$

where *i* runs over all positions in the genome. The probability that position *i* is sampled by the sequencer is denoted Pr(*i*), and is considered to be uniform both here and in [20]. The quantity Pr(*r*|*g*_*i*,…,*i* + *R*−1_) is the probability of observing the read *r* if the sequence at positions *i*,…,*i* + *R* − 1 in the genome is read by the sequencer. For reads that include base quality values *q*, let *q*_*i*_ denote the probability that a base in a read is incorrect. Then Pr(*r*|*g*) may be replaced by Pr(*r*|*gq*). In [20], Pr(*r*|*gq*) is rapidly approximated by summing the quality values of bases that mismatch in the ungapped alignment between *r* and *g*_*i*,…,*i* + *R* − 1_. When there are insertions and deletions in the sequence, the value Pr(*r*|*g*_*i*,…,*i* + *R* − 1_) may be computed as $\left(\underset{f}{\text{Pr}}(r|g_{i,\ldots ,i+R-1},H)\right)$; this denotes the forward algorithm probability using a pairwise hidden Markov model (Pair-HMM) $\left(H\right)$ that encodes probabilities for substitution, insertion, and deletion at every position.

The denominator of Equation 1 gives the marginal probability that the read is observed from anywhere in the genome. Evaluating this full sum is computationally infeasible even for short reads and ungapped alignments. Since the probability of observing a read given a template sequence drops geometrically with divergence, most positions in the genome do not contribute significantly to the sum. For short reads, the sum is approximated in [20] as the sum of the probability of the top scoring alignment and all second best alignments.

In BLASR, the mapping quality value is calculated in a similar manner. The sum in Equation 1 is limited to the top MaxCandidates alignments, and is then scaled by a factor that reflects the limited sample size by aligning only at most MaxCandidates clusters. When the read is sampled from a unique region of the genome, there will be few clusters of high score, and the highest scoring cluster will likely contain the true match to the genome. However, when the read is sampled entirely from a repetitive sequence, there will be many high scoripng clusters. In this case, it is possible the cluster from the correct interval on the genome will not have high enough score to be retained in MaxCandidates clusters. To account for this, we assume that the correct interval in the genome may correspond to any significantly highly scoring cluster, and multiply the sum in Equation 1 by the ratio of the number of significant clusters found in the genome to MaxCandidates, as long as the number of significantly highly scoring clusters is greater than MaxCandidates. The significance of a cluster may be measured by comparing the number of anchors in a cluster to the number of anchors expected at the correctly mapped location. The distributions of numbers of anchors expected to correctly map were found using simulations of error processes for different error rates and read lengths; however, it is possible to model this theoretically (see the next section). The expected number of anchors a read has when mapped to the correct location is genome-independent: it depends only on the error rate, length of the read, and minimum anchor length. We use a slightly different metric, the number of anchor-bases (the total number of bases in all anchors) to measure cluster significance, and this is similarly genome-independent. For efficiency, in BLASR we precompute the expectation and variance for the number of anchor-bases for a range of feasible accuracies, read lengths, and minimum match lengths, and minimum match size. The accuracy of the highest scoring alignment is used as a proxy for the true accuracy of the read. Given the accuracy, the length of the aligned sequence, and the minimum match length, we look up the mean *μ* and variance *σ*^2^ for number of anchor bases, and count all clusters with more than *μ*−2*σ* anchor bases as significant.

## Appendix 1

### Enumeration of configurations with specified numbers of errors and anchors

In this section, we will show how to explicitly compute NumConfigurations(*M*,*N*,*K*,*L*).

Consider a read of length *L* with exactly *M* errors, at positions 1 ≤ *x*_1_ < *x*_2_ < … < *x*_*M*_ ≤ *L*. Also set *x*_0_ = 0 and *x*_*M* + 1_ = *L* + 1.

For the sake of simplicity, we assume all sequencing errors are of length 1, but this can be generalized to insertions and deletions that change the length of the read.

The error positions split the read into **parts** of sizes *λ* = *x*_*i*_ − *x*_*i*−1_ ≥ 1 for *i* = 1,…,*M* + 1. Each part *λ*_*i*_ (*i* = 1,…,*M*) consists of *λ*_*i*_ − 1 matches followed by one mismatch. The last part consists of *λ*_*M* + 1_ − 1 matches. Note that if there are two consecutive mismatches, there will be a part *λ*_*i*_ = 1 corresponding to 0 matches followed by one mismatch.

Part sizes *λ*_*i*_ are related to the notation *W * of the Results section by *λ*_*i*_ = *W* + 1. Note that *W * counted only the correct positions, and we did not have a subscript (*W*_*i*_) to specify the word number. In this section, *λ*_*i*_ counts the correct bases and also counts one incorrect base at the end, based on our simplification that all sequencing errors are of length one.

Set *λ* = (*λ*_1_,*λ*_2_,…,*λ*_*M* + 1_). These are positive integers that add up to *L* + 1. In Combinatorics, this is called a **strict composition** of *L* + 1 into *M* + 1 parts. Let *K* be the minimum anchor length (a parameter).

Consecutive errors greater than *K* apart (*λ*_*i*_ > *K*) give segments that are **anchors** while consecutive errors shorter than this (*λ* ≤ *K*) give segments called **short matches**.

**Figure 10:**
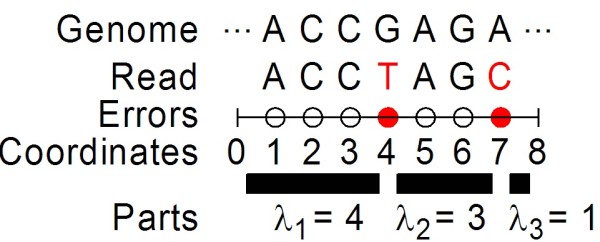
**Toy example for counting components.** A read of length *L* = 7 with *M* = 2 errors is shown, with errors in red. In general, *M* errors splits the read into *M* + 1 parts, some of which may be null; in this case, the third part is null. For anchor length threshold *K* = 3 (meaning parts of size >3 are anchors, parts of size ≤3 are not), we have *N* = 1 anchor (the first part).

In Figure 10, we illustrate a read of length *L* = 7 with *M* = 2 error positions. For a minimum anchor length *K* = 3, there are 6 compositions where the first part is an anchor:

(4,3,1),(4,2,2),(4,1,3),(5,2,1),(5,1,2),(6,1,1).

For reads of length 7 with 2 errors, and minimum anchor length 3, the number of compositions with exactly one anchor (allowing it to be any of the parts, via permutations of these compositions) is 6·3 = 18.

For arbitrary values of the parameters, we first compute the number of configurations where all *N* anchors come first and all *M* + 1−*N* short parts come last. Then we multiply the count of these by M+1N to allow any of the *N* parts to be the anchors.

Let *N* ≥ 0 be an integer. For given parameters *M*,*N*,*K*,*L*, we will enumerate the number of arrangements of error positions that result in exactly *N* anchors. This is equivalent to the combinatorial problem of counting **integer compositions** of *L* + 1 with certain restrictions on the sizes of the parts. We will use generating function techniques from combinatorics to count arrangements of *M* error positions that give exactly *N* anchors (so the other *N* + 1−*M* parts are short fragments). Let *c*_*M*,*N*,*K*_(*L*) denote the number of arrangements of *M* error positions that result in exactly *N* anchors, where the read length is *L* and anchors are defined as parts *λ*_*i*_ > *K*. Let $\left(c_{M,N,K}^{\prime }(L)\right)$ be the number of arrangements where all the anchors precede all the short parts (*λ*_1_,…,*λ*_*N*_ > *K*) and *λ*_*N* + 1_,…,*λ*_*M* + 1_ ≤ *K*). These are related by cM,N,K(L)=M+1NcM,N,K′(L) since we can select any *N* of the *M* + 1 parts to be the anchors.

Note that

(A1) $$\begin{array}{ll} \text{NumConfigurations}(M,N,K,L) & =\underset{N^{\prime }\geq N}{\sum }c_{M,N^{\prime },K}(L)\; \end{array}$$

The compositions of *L* + 1 into *M* + 1 parts, where the first *N* parts are anchors and the remaining *M* + 1 − *N* parts are short, have the following constraints: 

• *λ*_1_,…,*λ*_*N*_ ∈ 1,2,…,*K*(short parts).

• *λ*_*N* + 1_,*λ*_*N* + 2_,…,*λ*_*M*_ ∈ *K* + 1,*K* + 2,… (anchors).

• *λ*_1_ + ⋯ + *λ*_*M*_ = *L* + 1.

The generating functions for short parts, *S*(*t*), and anchors, *A*(*t*), are

(A2) $$\begin{array}{ll} S(t)=t^{1}+t^{2}+\cdots +t^{K}=\frac{t(1-t^{K})}{1-t} & \; \end{array}$$

(A3) $$\begin{array}{ll} A(t)=t^{K+1}+t^{K+2}+\cdots =\frac{t^{K+1}}{1-t} & \; \end{array}$$

Standard methods for enumerating compositions with generating functions give that

(A4) $$A{\left(t\right)}^{N}S{\left(t\right)}^{M+1-N}=\underset{L}{\sum }c_{M,N,K}^{\prime }(L)t^{L+1}$$

where we expand the left side in a MacLaurin series (Taylor series centered at *t* = 0) to obtain the right side. The counts $\left(c_{M,N,K}^{\prime }(L)\right)$ are the coefficients in this series. Multiplying this by M+1N gives

(A5) GM,N,K(t)=M+1NA(t)NS(t)M+1−N=∑LcM,N,K(L)tL+1

To compute *c*_*M*,*N*,*K*_(*L*), we use Taylor series methods to compute the coefficient of *t*^*L* + 1^in (A5). We present two methods to do this.

**First Taylor series method:** The coefficient of *t*in (A5) may be determined by polynomial multiplication. We truncate the middle expression in (A3) to terms of degree ≤ *L* + 1, which turns it into a polynomial; the middle expression of (A2) is already a polynomial. We take powers and products of the polynomials, truncating terms of degree > *L* + 1 at intermediate steps. The coefficient of *t*^*L* + 1^ in the result is *c*_*M*,*N*,*K*_(*L*). All intermediate products and sums involve only nonnegative integers.

**Second Taylor series method:** We present an exact closed-form solution. Mathematically, closed-form solutions are usually preferred. However, the first method above may be preferable for computation because intermediate steps of this second method require much higher precision, as discussed in Appendix 2.

#### Theorem A1

*ForK* = 0: if *N* = *M* + 1 *then*cM,N,K(L)=M+1N; *otherwise,c*_*M*,*N*,*K*_(*L*) = 0.

*ForK* ≥ 1, *setD* = *L* − *NK* − *Mandi*_max_ = min(⌈*D*/*K*),*M* + 1−*N*⌉.

*IfD* < 0 *ori*_max_ < 0 *thenc*_*M*,*N*,*K*_(*L*) = 0. *Otherwise,*

(A6) cM,N,K(L)=M+1N∑i=0imax(−1)iM+1−NiM+D−iKM.

#### Proof

For *K*=0, there are no short parts; all parts are anchors. This is equivalent to counting the number of strict compositions of *L* + 1 into *M* + 1 parts, which is well-known to be LM.

For *K* ≥ 1, note that we may write

(A7) GM,N,K(t)=M+1NA(t)NS(t)M+1−N=M+1NtKN+M+1(1−tK)M+1−N(1−t)M+1

The binomial theorem and the negative binomial series give

(1−tK)M+1−N=∑i=0M+1−N(−1)iM+1−NitiK1(1−t)M+1=∑j=0∞M+jMtj

Plugging these into (A7), we obtain

(A8) GM,N,K(t)=M+1N∑i=0M+1−N∑j=0∞M+jM(−1)i×M+1−NitKN+(M+1)+iK+j

In (A5), the coefficient of *t*^*L* + 1^ is *c*_*M*,*N*,*K*_(*L*). Collecting together the terms in (A8) where the exponent of *t* is *L* + 1 gives (A6). We omit the detailed but straightforward derivation. □

## Appendix 2

### Numerical precision of the closed form solution for the number of anchors

Theorem A1 (also called the “Second Taylor series method”) gives a closed form expression (A6) to compute *c*_*M*,*N*,*K*_(*L*). This closed form solution has only a small number of terms. However, for practical parameter values, it may require more bits of precision than are available in a finite precision computation, even if the final answer does not overflow the variable size. This is because the expression has an alternating sum with terms of much higher absolute value than the final answer. Consider this part of the summation in Theorem A1, omitting the coefficient M+1N:

∑i=0imax(−1)iM+1−NiM+D−iKM.

For *M* = 75, *N* = 1, *K* = 15, *L* = 1000, this has 61 alternating terms of magnitude between 2^93^ and 2^401^, while the value of the sum is much smaller, with magnitude 2^294^. Using high precision floating point, we need at least 110 bits for the mantissa to get the first decimal digit correct. This is significantly more bits than is currently standard: the current standard for floating point, IEEE 754, provides for a 53 bit mantissa in double precision. Alternatively, using high precision integers, we would need 294 bits of integer precision, plus a sign bit. However, software for arbitrary precision integers, such as Maple or Mathematica, will handle this example correctly.

By contrast, the “First Taylor series method” only involves sums and products of positive integers, each bounded above by the value of *c*_*M*,*N*,*K*_(*L*). Thus, if the integer precision is adequate to store the value of *c*_*M*,*N*,*K*_(*L*), it is also adequate to perform all intermediate calculations.

## Appendix 3

### Statistics of number of anchors

We may estimate the number of anchors using the following theorem.

#### Theorem A2

Fix *M*,*K*,*L*. Under the uniform distribution on compositions of *L* + 1 into *M* + 1 parts, the mean number of anchors and its variance are given by

(A9) μ=E[N]=(M+1)L−KMLM

(A10) σ2=Var[N]=M(M+1)L−2KMLM+(M+1)L−KMLM−(M+1)L−KMLM2.

For fixed *M* and *K*, consider the two-variable generating function

(A11) $$\begin{array}{ccc} H_{M,K}(t,u) & = & \overset{\infty }{\underset{N=0}{\sum }}\underset{L}{\sum }c_{M,N,K}(L)t^{L+1}u^{N}\;\\ & = & \overset{\infty }{\underset{N=0}{\sum }}G_{M,N,K}(t)u^{N}\; \end{array}$$

(A12) =∑N=0∞M+1NtK+11−tNuN×t(1−tK)1−tM+1−N=utK+11−t+t(1−tK)1−tM+1=(u−1)tK+1+t1−tM+1

For fixed *M*,*K*,*L*, the probability of exactly *N* anchors is *c*_*M*,*N*,*K*_(*L*)/*T*, where

$$T=\overset{\infty }{\underset{N^{\prime }=0}{\sum }}c_{M,N^{\prime },K}\left(L\right)$$

Note that *T* counts the total number of compositions of *L* + 1 into *M* + 1 parts, with 0 or more anchors. Thus, it actually counts the total number of compositions of *L* + 1 into *M* + 1 parts, without regard to sizes of parts. So we have:

(A13) T=∑N′=0∞cM,N′,K(L)=LM

and thus the probability of exactly *N* anchors is cM,N,K(L)/LM.

Next, for fixed *M*,*K*,*L*, we evaluate *E*[*N*], the mean number of anchors under the uniform distribution of compositions of *L* + 1 into *M* + 1 parts.

E[N]=∑NcM,N,K(L)LM·N

Using standard generating function properties, the numerator $\left(\underset{N}{\sum }c_{M,N,K}(L)\cdot N\right)$ is the coefficient of *t*^*L* + 1^ in the following expression:

(A14) $${\left(\frac{\partial }{\mathrm{\partial u}}H_{M,K}(t,u)\right|}_{u=1}$$

First we evaluate the derivative; second, we plug in *u* = 1; third, we extract the coefficient of *t*^*L* + 1^; and fourth, we use this to compute *E*[*N*]: 

1. The derivative in Eq. (A14) is

$$\begin{array}{lll} \frac{\partial }{\mathrm{\partial u}}H_{M,K}(t,u) & =\frac{\partial }{\mathrm{\partial u}}{\left(\frac{(u-1)t^{K+1}+t}{1-t}\right)}^{M+1}\; & \;\\ & =(M\;+\;1){\left(\frac{(u\;-\;1)t^{K+1}+t}{1-t}\right)}^{M}\frac{t^{K+1}}{1-t}\; & \; \end{array}$$

2. Plug in *u* = 1:

$$\begin{array}{lll} {\left(\frac{\partial }{\mathrm{\partial u}}H_{M,K}(t,u)\right|}_{u=1} & =(M+1){\left(\frac{t}{1-t}\right)}^{M}\frac{t^{K+1}}{1-t}\; & \;\\ & =(M+1)\frac{t^{M+K+1}}{{\left(1-t\right)}^{M+1}}\; & \; \end{array}$$

3. Expand the Taylor series and extract the coefficient of *t*^*L* + 1^:

(M+1)tM+K+1(1−t)M+1=(M+1)∑j=0∞M+jMtM+K+1+j

The term *t*^*L* + 1^occurs when *j* = *L* − *M* − *K*.If *j* < 0, this coefficient is 0. If *j* ≥ 0, this coefficient is (M+1)L−KM.

4.Evaluate *E*[*N*] to obtain Equation (A9):

E[N]=(M+1)L−KMLM.

Note that if *L* − *K* < *M*, then *E*[*N*] = 0.

Next we compute the variance of N, using a similar generating function technique. The generating function will enable us to compute *E*[*N*(*N* − 1)], so we will compute the variance in the form

$$\sigma^{2}=E[N(N-1)]+E[N]-E{\left[N\right]}^{2}$$

which is equivalent to the more common formula *σ*^2^ = *E*[*N*^2^] − *E*[*N*]^2^. We have:

E[N(N−1)]=∑NcM,N,K(L)LM·N(N−1)

The numerator $\left(\underset{N}{\sum }c_{M,N,K}(L)\cdot N(N-1)\right)$ is the coefficient of *t*^*L* + 1^in the following expression:

(A15) $${\left(\frac{\partial^{2}}{\partial u^{2}}H_{M,K}(t,u)\right|}_{u=1}$$

We evaluate this in a fashion similar to *E*[*N*]: 

1. The derivative in Eq. (A15) is

$$\begin{array}{lll} \frac{\partial^{2}}{\partial u^{2}}H_{M,K}(t,u) & =\frac{\partial^{2}}{\partial u^{2}}{\left(\frac{(u-1)t^{K+1}+t}{1-t}\right)}^{M+1}\; & \;\\ & =M(M+1){\left(\frac{(u-1)t^{K+1}+t}{1-t}\right)}^{M-1}\; & \;\\ & \;\times {\left(\frac{t^{K+1}}{1-t}\right)}^{2}\; & \; \end{array}$$

2. Plug in *u* = 1:

$$\begin{array}{lll} {\left(\frac{\partial^{2}}{\partial u^{2}}H_{M,K}(t,u)\right|}_{u=1} & \;=\;M(M\;+\;1){\left(\;\frac{t}{1\;-\;t}\;\right)}^{M-1}{\left(\;\frac{t^{K+1}}{\;-\;t}\;\right)}^{2}\; & \;\\ & =M(M+1)\frac{t^{M+1+2K}}{{\left(1-t\right)}^{M+1}}\; & \; \end{array}$$

3. Expand the Taylor series and extract the coefficient of *t*^*L* + 1^:

M(M+1)tM+1+2K(1−t)M+1=M(M+1)tM+1+2K∑j=0∞M+jMtj=M(M+1)∑j=0∞M+jMtM+1+2K+j

The term *t*^*L* + 1^ occurs when *j* = *L* − (*M* + 2*K*). This coefficient is M(M+1)L−2KM (which is 0 when *L* − 2*K* < *M*).

4. Evaluate *E*[*N*(*N* − 1)]:

E[N(N−1)]=M(M+1)L−2KMLM

5. Evaluate *σ*^2^ = Var[*N*] to prove Equation (A10):

σ2=Var[N]=E[N(N−1)]+E[N]−E[N]2=M(M+1)L−2KMLM+(M+1)L−KMLM−(M+1)L−KMLM2

## Appendix 4

### Asymptotic number of anchors

#### Theorem A3

Let *μ*,*σ*^2^ be given by Theorem A2. For sufficiently large *M*,

(A16) cM,N,K(L)≈LMϕN−μσ

(A17) NumConfigurations(M,N,K,L)≈LM×1−ΦN−12−μσ

where $\left(\phi (z)=\frac{1}{\sqrt{2\Pi }}e^{-z^{2}/2}\right)$ and *Φ*(*z*) are the probability density function and cumulative distribution function of the standard normal distribution.

#### Proof

For fixed *M*,*K*, Eq. (A12) gives the generating function *H*_*M*,*K*_(*t*,*u*) as a rational function in *t*,*u*, raised to the power *M* + 1. When *M* is sufficiently large, the Central Limit Theorem gives that the coefficients *c*_*M*,*N*,*K*_(*L*) in its Taylor series, Eq. (A11), are well-approximated by a bivariate normal distribution. Restricting to the coefficients of *t*^*L* + 1^ for fixed *L* gives that the coefficients of $\left(f(u)=\overset{\infty }{\underset{N=0}{\sum }}c_{M,N,K}(L)u^{N}\right)$ are approximated by a univariate normal distribution. This represents the distribution of *N* for fixed *M*,*K*,*L*. We computed the parameters *μ*,*σ*^2^of this distribution in Theorem A2. By Eq. (A13), the total of the coefficients in *f*(*u*) is f(1)=T=LM. Note that $\left(\text{NumConfigurations}(M,N,K,L)\right)$ is the survival function of *c*_*M*,*N*,*K*_(*L*):

$$\begin{array}{lll} \text{NumConfigurations}(M,N,K,L) & =\underset{N^{\prime }\geq N}{\sum }c_{M,N^{\prime },K}(L)\;.\; & \; \end{array}$$

Thus, we obtain Eqs. (A16) and (A17) as approximations for the coefficients *c*_*M*,*N*,*K*_(*L*) and the survival function NumConfigurations(*M*,*N*,*K*,*L*). In Eq. (A17), note that $\left(-\frac{1}{2}\right)$ is a continuity correction. □

**Figure 11:**
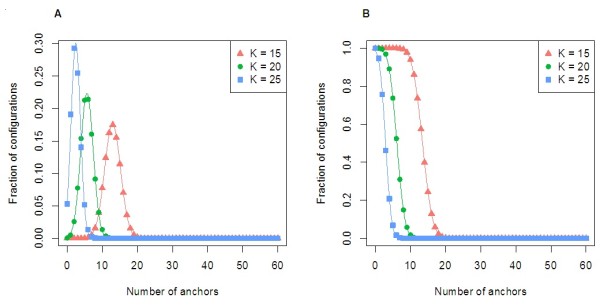
**The fraction of configurations with exactly and at least*N*anchors.** (**A**) Plot of the fraction of configurations with exactly *N* anchors, cM,N,K(L)/LM, as *N* varies. An anchor is a run of at least *K* correct bases (shown for *K* = 15, 20, and 25). We assume the read length is *L* = 1000 and the error rate per base is *ρ* = 15*%*(and that there are exactly *M* = 150 error positions). The solid markers are computed by finding exact coefficients *c*_*M*,*N*,*K*_(*L*) in the generating functions. The curve is a normal distribution approximating the exact values (illustrating Theorem A3), where parameters *μ*and *σ*^2^are computed by Theorem A2. (**B**) The solid markers are a plot of NumConfigurations(M,N,K,L)/LM, the fraction of configurations with at least *N* anchors, as *N* varies. The parameters are the same as for (A). The curve is the survival function of the normal distribution in (A).

In Figure 11, we plot *c*_*M*,*N*,*K*_(*L*) and NumConfigurations(M,N,K,L). The solid markers are the true values computed from the generating function. The curve is the estimate computed by the preceding theorem, and does indeed approximate the true values well.

## Competing interests

MJC is a full-time employee at Pacific Biosciences, a company commercializing single-molecule, real-time nucleic acid sequencing technologies. GT was partially supported by a grant from the National Institutes of Health, USA (NIH grant 3P41RR024851-02S1). NIH had no role in study design, data collection and analysis, decision to publish, or preparation of the manuscript.

## Author’s contributions

MJC proposed and implemented the mapping method, performed the analysis, and wrote the manuscript. GT solved and implemented the combinatorial analysis, and wrote the manuscript. Both authors read and approved the final manuscript.

## Supplementary Material

Additional file 1 **Supplementary Text S1.** The supplementary text contains additional implementation details for the anchor similarity method, and description of the empirical model based read simulator. Click here for file

Additional file 2 **Supplementary Table S1.** Supplementary Table S1 gives the command line parameters used to run the benchmarks. Click here for file

## References

[B1] Smith T Waterman M Identification of Common Molecular Subsequences J Mol Biol 1981 147 194 197 10.1016/0022-2836(81)90087-5 7265238

[B2] Zhang Z Schwartz S Wagner L Miller W A greedy algorithm for aligning DNA sequences J Comput Biol 2000 7 203 214 10.1089/10665270050081478 10890397

[B3] Kent W BLAT–the BLAST-like alignment tool Genome Res 2002 12 656 664 11932250 10.1101/gr.229202 PMC187518

[B4] Langmead B Trapnell C Pop M Salzberg S Ultrafast and memory-efficient alignment of short DNA sequences to the human genome Genome Biol 2009 10 R25 10.1186/gb-2009-10-3-r25 19261174 PMC2690996

[B5] Li H Durbin R Fast and accurate short read alignment with Burrows-Wheeler transform Bioinformatics 2009 25 1754 1760 10.1093/bioinformatics/btp324 19451168 PMC2705234

[B6] Li R Yu C Li Y Lam T Yiu S Kristiansen K Wang J SOAP2: an improved ultrafast tool for short read alignment Bioinformatics 2009 25 1966 1967 10.1093/bioinformatics/btp336 19497933

[B7] Ferragina P Manzini G Opportunistic data structures with applications Proc. of the 41st IEEE Symp on Found of Comput Sci 2000 390 398

[B8] Rasko D Webster D Sahl J Bashir A Boisen N Scheutz F Paxinos E Sebra R Chin C Iliopoulos D Klammer A Peluso P Lee L Kislyuk A Bullard J Kasarskis A Wang S Eid J Rank D Redman J Steyert S Frimodt-Mller J Struve C Petersen A Krogfelt K Nataro J Schadt E Waldor M Origins of the E. coli strain causing an outbreak of hemolytic-uremic syndrome in Germany New England J Med 2011 365 709 717 10.1056/NEJMoa1106920 21793740 PMC3168948

[B9] Brudno M Poliakov A Salamov A Cooper G Sidow A Rubin E Solovyev V Batzoglou S Dubchak I Automated Whole-Genome Multiple Alignment of Rat, Mouse, and Human Genome Res 2004 14 685 692 10.1101/gr.2067704 15060011 PMC383314

[B10] Schwartz S Kent W Smit A Zhang Z Baertsch R Hardison R Haussler D Miller Human-Mouse Alignments with BLASTZ Genome Res 2003 13 103 107 10.1101/gr.809403 12529312 PMC430961

[B11] Kurtz S Phillippy A Delcher A Smoot M Shumway M Antonescu C Salzberg S Versatile and open software for comparing large genomes Genome Biol 2004 5 R122 R129 10.1186/gb-2004-5-2-r12 PMC395750 14759262

[B12] Altschul S Gish W Miller W Myers E Lipman D Basic local alignment search tool J Mol Biol 1990 215 403 410 2231712 10.1016/S0022-2836(05)80360-2

[B13] Lipman D Pearson W Rapid and sensitive protein similarity searches Science 1985 4693 1435 1441 2983426 10.1126/science.2983426

[B14] Bray N Dubchak I Pachter L AVID: A global alignment program Genome Res 2003 13 97 102 10.1101/gr.789803 12529311 PMC430967

[B15] Darling A Mau B Blatter F Perna N Mauve: multiple alignment of conserved genomic sequence with rearrangements Genome Res 2004 14 1394 1403 10.1101/gr.2289704 15231754 PMC442156

[B16] Brudno M Do C Cooper G Kim M Davydov E Green E Sidow A Batzoglou S LAGAN and Multi-LAGAN: efficient tools for large-scale multiple alignment of genomic DNA Genome Res 2003 13 721 731 10.1101/gr.926603 12654723 PMC430158

[B17] Kent WJ Baertsch R Hinrichs A Miller W Haussler D Evolution’s cauldron: Duplication, deletion, and rearrangement in the mouse and human genomes Proc Nat Acad Sci 2003 100 11484 11489 10.1073/pnas.1932072100 14500911 PMC208784

[B18] Li H Durbin R Fast and accurate long-read alignment with Burrows Wheeler transform Bioinformatics 2010 26 589 595 10.1093/bioinformatics/btp698 20080505 PMC2828108

[B19] Li R Li Y Kristiansen K Wang J SOAP: short oligonucleotide alignment program Bioinformatics 2008 24 713 714 10.1093/bioinformatics/btn025 18227114

[B20] Li H Ruan J R D Mapping short DNA sequencing reads and calling variants using mapping quality scores Genome Res 2008 18 1851 1858 10.1101/gr.078212.108 18714091 PMC2577856

[B21] Rumble S Lacroute P Dalca A Fiume M Sidow A SHRiMP: Accurate Mapping of Short Color-space Reads PLoS Comput Biol 2009 5 e1000386 10.1371/journal.pcbi.1000386 19461883 PMC2678294

[B22] Durbin R Eddy S Krobh A Mitchison G Biol Sequence Anal: Probabilistic Models of Proteins and Nucleic Acids. The Edinburgh Building, Cambridge, CB2 2RU 1998 United Kingdom: Cambridge University Press

[B23] Needleman S Wunsch C A general method applicable to the search for similarities in the amino acid sequence of two proteins J Mol Biol 1970 48 443 453 10.1016/0022-2836(70)90057-4 5420325

[B24] Eid J Fehr A Korlach J Turner S Real-time DNA sequencing from single polymerase molecules Science 2009 323 133 138 10.1126/science.1162986 19023044

[B25] Clarke J Wu H Jayasinghe L Patel A Reid S Bayley H Continuous base identification for single-molecule nanopore DNA sequencing Nat Nanotechnol 2009 4 265 270 10.1038/nnano.2009.12 19350039

[B26] Cherf G Lieberman K Rashid H Lam C Karplus K Akeson M Automated forward and reverse ratcheting of DNA in a nanopore at 5-Å precision Nat Nanotechnol 2012 14 344 348 10.1038/nbt.2147 PMC3408072 22334048

[B27] Garaj S Hubbard W Reina A Kong J Branton D Golovchenko J Graphene as a subnanometre trans-electrode membrane Nature 2010 467 190 193 10.1038/nature09379 20720538 PMC2956266

[B28] Altschul S Madden T Schäffer A Zhang J Zhang Z Miller W Lipman D Gapped BLAST and PSI-BLAST: a new generation of protein database search programs Nucleic Acids Res 1997 17 3389 3402 9254694 10.1093/nar/25.17.3389 PMC146917

[B29] Weese D Emde A Rausch T Döring A Reinert K RazerS-fast read mapping with sensitivity control Genome Res 2009 19 1646 1654 10.1101/gr.088823.108 19592482 PMC2752123

[B30] Bao Z Eddy S Automated de novo identification of repeat sequence families in sequenced genomes Genome Res 2002 12 1269 1276 10.1101/gr.88502 12176934 PMC186642

[B31] Flusberg B Webster D Lee J Travers K Olivares E Clark T Korlach J SW T Direct detection of DNA methylation during single-molecule, real-time sequencing Nat Methods 2010 7 461 465 10.1038/nmeth.1459 20453866 PMC2879396

[B32] Alkan C Kidd J Marques-Bonet T Aksay G Antonacci F Hormozdiari F Kitzman J Baker C Malig M Mutlu O Sahinalp S Gibbs R Eichler E Personalized Copy-Number and Segmental Duplication Maps using Next-Generation Sequencing Nat Genet 2009 41 1061 1067 10.1038/ng.437 19718026 PMC2875196

[B33] Myers E Manber U Suffix arrays: A new method for on-line string searches SIAM J Comput 1993 22 935 948 10.1137/0222058

[B34] Abouelhoda M Ohlebusch E A Local Chaining Algorithm and its Applications in Comparitive Genomics Lecture Notes in Comput Sci 2812 2003 1 16

[B35] Eppstein D Galil Z Giancarlo R Italiano G Sparse Dynamic Programming I: Linear cost functions J Assoc Comput Machinery 1992 39 519 545 10.1145/146637.146650

