# Supplementary material for: Mapping single molecule sequencing reads using basic local alignment with successive refinement (BLASR): application and theory
Source: BMC Bioinformatics. 2012 Sep 19;13:238. doi: 10.1186/1471-2105-13-238 (PMC3572422; doi:10.1186/1471-2105-13-238)
Supplement: Additional file 1 — Supplementary Text S1. The supplementary text contains additional implementation details for the anchor similarity method, and description of the empirical model based read simulator. [file 1471-2105-13-238-S1.pdf]

Mapping single molecule sequencing reads using Basic Local Alignment with Successive Refinement (BLASR): Application and Theory

Mark J Chaisson[1] and Glenn Tesler[*2]

[1]Department Secondary Analysis, Pacific Biosciences, 1380 Willow Rd, Menlo Park, CA, 94025, USA
[2]Department of Mathematics, University of California, San Diego, 9500 Gilman Dr, La Jolla, CA, 92093, USA

Email: Glenn Tesler - gptesler@math.ucsd.edu;

[*]Corresponding author

Supplementary Text S1

1 Additional Implementation Details

1.1 Anchor similarity search

The anchor similarity of two sequences is the maximum number of shared words in the same order and orientation between the two sequences with limitations on the discrepancy of gap lengths between adjacent words between the two sequences. Given two sequences, r and g, let δ be a value that reflects the maximum insertion rate by a sequencer. In the case of the PacBioRS sequencer, $\delta = 0.15$. For every pair of adjacent anchors, let τ be the ratio of length separating the anchors in the first sequence to the length separating the anchors in the second. The anchor similarity is the maximum number of shared nonoverlapping words such that every gap ratio τ is constrained to $1 - \delta < \tau < 1 + \delta$.

To find the maximum number of anchors shared between two sequences, first find $\mathcal{A}^k$, the set of all matches of length k between the two sequences. Similarly to the definition of the global chaining, let $\text{Read}(a_i)$ and $\text{Genome}(a_i)$ be the starting position of anchor $a_i \in \mathcal{A}^k$ in the first sequence and second sequence respectively. The set of anchors $\mathcal{A}^k$ is sorted by $\text{Read}(a)$ and then by $\text{Genome}(a)$. Two arrays, PathCount and PathPrev, are defined

with size $|\mathcal{A}^k| + 1$. Define $\text{similar}(\mathcal{A}, i, j)$ to be a function indicating the ratio of the gaps between anchors falls between $1 - \delta$ and $1 + \delta$:

$$\text{similar}(\mathcal{A}, i, j) = \begin{cases} 1 & \text{if } j = 0 \text{ or } \dfrac{|(\text{Genome}(a_i) - \text{Read}(a_i)) - (\text{Genome}(a_j) - \text{Read}(a_j))|}{\min(\text{Read}(a_i) - \text{Read}(a_j), \text{Genome}(a_i) - \text{Genome}(a_j))} \leq \delta \\ 0 & \text{otherwise} \end{cases}.$$

The arrays PathCount and PathPrev are initiated with $\text{PathCount}[0] = 0$, and $\text{PathPrev}[0] = 0$, and then filled in for $i = 1, \ldots, |A^k|$ by using

$$\begin{aligned} \text{PathPrev}[i] &= \operatorname*{arg\,max}_{j \in \{0,\ldots,i-1\}} \text{PathCount}[j] \cdot \text{similar}(\mathcal{A}, i, j) \\ \text{PathCount}[i] &= \max_{j \in \{0,\ldots,i-1\}} \text{PathCount}[j] \cdot \text{similar}(\mathcal{A}, i, j)\,. \end{aligned}$$

When $i > 0$, the value $l = \text{PathCount}[i]$ is the maximum number of anchors among $\{a_1, a_2, \ldots, a_i\}$ shared between the two sequences, assuming anchor a_i is the final anchor in such a chain of anchors.

Let i^* be the index with maximum value of $\text{PathCount}[i^*]$; if there's a tie, take the minimum index achieving the maximum. The value of $\text{PathCount}[i^*]$ is the anchor similarity.

The maximal set of anchors shared between the sequences is $\{a_{i_1}, a_{i_2}, \ldots, a_{i_l}\}$ is found by backtracking with the PathPrev array: set $i_1 = i^*$ and iterate $i_{r+1} = \text{PathPrev}[i_r]$. At termination, note that $i_l > 0$ and $i_{l+1} = 0$.

Although this method runs in $O(n^2)$ time, as opposed to the other global chaining methods that run in $O(n \log n)$ time, it was sufficient for short (1000-base) intervals in this study.

1.2 Empirical read simulator

The model contains two components: (1) a read length model, and (2) a base and quality value output model, both of which are derived from alignments. The length model is simply a collection of all lengths of aligned reads, plus four extra bases to account for how bases are generated. Simulated read lengths are sampled uniformly from this collection. For all 4^5 five base sequence contexts, 2000 samples of the bases and quality values are output for the

middle base; these are tallied by scanning though sample alignments. To produce simulated reads, a target sequence is selected with a random position uniformly sampled from the genome with a length sampled uniformly from the aligned sequence length collection, plus an extra eight bases. To produce the sequence of the read and all associated quality values, the target sequence is scanned and for all 5-mers, a sample output is selected uniformly from the collection of output stored for that context. No output is produced for the two bases at the beginning and end of the target sequence.

1.3 Method parameters

To run benchmarks, methods were supplied with the parameters in Table S1 and the same input files.
