# Supplementary material for: Mapping single molecule sequencing reads using basic local alignment with successive refinement (BLASR): application and theory
Source: BMC Bioinformatics. 2012 Sep 19;13:238. doi: 10.1186/1471-2105-13-238 (PMC3572422; doi:10.1186/1471-2105-13-238)
Supplement: Additional file 2 — Supplementary Table S1. Supplementary Table S1 gives the command line parameters used to run the benchmarks. [file 1471-2105-13-238-S2.pdf]

Table S1. Parameters for each alignment program

Method	Parameters
BLASR	-sam -bestn 1
BWA-SW	-b5 -q2 -r1 -z10
BLAT	-t=dna -q=dna -stepSize=1
